# Decompression induces inflammation but do not modify cell proliferation and apoptosis in odontogenic keratocyst

**DOI:** 10.4317/jced.59096

**Published:** 2022-01-01

**Authors:** Daniela Trujillo-González, Mariana Villarroel-Dorrego, Raiza Toro, Gabriela Vigil, Vanesa Pereira-Prado, Ronell Bologna-Molina

**Affiliations:** 1 Universidad Central de Venezuela, Caracas, Venezuela; 2 Área de Patología Molecular Estomatológica, Facultad de Odontología, UdelaR, Montevideo-Uruguay

## Abstract

**Background:**

Odontogenic keratocyst (OKC) is a development cyst, of odontogenic origin, that differs from other entities by its infiltrating and aggressive biological behavior. Among conservative treatments for large lesions, surgical decompression stands out, with a variable recurrence rate. Aim: To evaluate the histological effects of decompression treatment on OKC, including cell proliferation and apoptosis of epithelial cyst.

**Material and Methods:**

21 OKC cases were included. Samples were taken before and after surgical decompression for histological evaluation and immunohistochemical staining of Ki-67, MCM4/7, Bax and Bcl2. Data were analyzed and compared using Student’s t and Wilcoxon tests for related samples, and p values <0,05 were considered statistically significant.

**Results:**

After decompression treatment an increase in inflammation of the cystic wall (*p*=0,029), loss of parakeratinization of the epithelium (*p*=0,007) and absence of palisade cell distribution in the basal layer were observed (*p*=0,002). There were no statistically significant changes in the expression of Ki-67 (*p*=0,323), MCM4/7 (*p*=0,079), Bax (*p*=0,392) or Bcl-2 when compared before and after decompression.

**Conclusions:**

Surgical decompression generates histological structural changes in OKC both in the epithelium and connective wall, however, these findings do not seem to alter induction of the cell cycle or epithelial apoptosis.

** Key words:**Odontogenic keratocyst, MCM, Bax, Bcl2, Ki-67, apoptosis, decompression.

## Introduction

As described by the World Health Organization (1), odontogenic keratocyst (OKC) is a development odontogenic cyst characterized by a thin, regular lining of parakeratinized stratified squamous epithelium. OKCs account for 10-20% of odontogenic cysts and are the third most common cyst of the jaws. OKC occurs over a wide patient age range, with a peak incidence in the second to third decades of life and a second peak among patients aged 50-70 years (1).

OKC differs from other developmental odontogenic cysts due to its infiltrative and aggressive biological behavior, associated to an increased recurrence rate. Recurrence is attributed to various factors (1-4), for instance thickness of the cyst wall, which can easily separate from the underlying connective tissue, which could contribute to disperse the epithelium fragments within the surrounding tissue and, still debatable, the presumption that the cystic epithelium has the capacity of independent growth (2,3).

Treatment modalities are varied, among the conservative options, surgical decompression is a popular technique, which creates a communication between the cystic cavity and the oral environment through a device in order to relieve the internal pressure within the cyst, and therefore, the size and volume of the lesion, allowing the preservation of adjacent vital structures (2-4). Additionally, decompression generates a thickening of the fibrous capsule of the cyst, which facilitates the total excision of the lesion (5,6). However, the main disadvantage of decompression is the high rate of recurrence (2-4).

Studies that describe the influence of decompression OKC treatment from a histological and immunohistochemical point of view and the changes that may occur are limited. Moreover, increased recurrence rates have been observed after marsupialization or decompression when are performed without any adjuvant treatments (3,4). Tabrizi *et al*. (4) hypothesized that creating a large cystic communication promotes histological changes which may induce multiple daughter cyst distribution throughout the wall and consequently augmented recurrence rate. Therefore, the purpose of this study was to evaluate the tissular and cellular effects of decompression on OKC epithelial lining with special interest in cell proliferation.

## Material and Methods

-Study sample 

A total of 42 samples were taken from 21 patients diagnosed with OKC and treated using surgical decompression. Both, before and after decompression samples were included for comparison. The study was conducted in accordance with the ethical standards of the Helsinki Declaration, and was approved by the Bioethics committee of Dental School, Universidad Central de Venezuela (CB-084-2019).

-Histopathology and immunohistochemistry 

Morphological changes of OKC were evaluated by H&E staining. Sections of 4µm were cut for indirect biotin-streptavidin-peroxidase staining. For antigen retrieval, Bulleye Decloaker (Biocare Medical) in a pressure cooker in microwave at a maximum pressure (750W) for 5 minutes was used. Tissue sections were incubated with Ki-67 (DAKO® clone M1B-1, 1:100 dilution), mini-chromosome maintenance protein (MCM) MCM4 (Abcam, Anti-MCM4 antibody, clone ab88546 mouse polyclonal. 1:100 dilution), MCM7 (Abnova clone DCS-141.1, 1:100 dilution), Bcl2 (Biocare clone 100/D5, 1:100 dilution) and polyclonal Bax (DAKO®, 1:200 dilution) antibodies. Detection and amplification system Envision (DAKO®) was applied for 30 minutes. Slices were exposed to 3’-diaminobenzidine-H2O2 (DAKO®) and finally stained with Mayer’s hematoxylin.

-Ki-67 and MCM4/7 cell quantification

Nuclear immunoreactivities were assessed as described previously (7). A 40x microphotograph per field (five fields per case) was taken using a light microscope (Olympus®). Images were transferred to a grid and saved as JPEG files. The count of positive cells was made using the digital image processing software ImageJ (1.46ª version).

-Bax and Bcl-2 cell quantification

Bax and Bcl-2 positivity was evaluated semi-quantitatively by cytoplasmic cell expression in the epithelial cyst. When percentage of immunopositive cells were equal to zero, it was cataloged as negative, less than 25% was categorized with one + (mild), between 25 and 50% with ++ (moderate) and, finally equal or greater than 50% with +++ (intense).

-Statistical analysis

Data obtained were analyzed using mean ± standard deviations (SD) in addition to frequencies and percentages. Quantitative variables were compared using Student t-test for related samples, assuming a normal distribution. Qualitative variables were compared using Wilcoxon test for paired samples. Comparisons were considered statistically significant if *p* value was less than 0,05.

## Results

A total of 21 patients were included in this study, distributed in 13 women (61, 9%) and 8 men (38, 1%). The mean age was 27, 33±14, 9 years, with a minimum age of 9 years and a maximum age of 58 years.

Of the 21 cases evaluated, most were found in the mandible, all 13 cases located in the posterior area (61,9%), the rest were situated in the maxilla, 6 cases in the anterior area (28,6%) and 2 in the posterior area (9,5%). No cases were found in the anterior mandibular area.

Between the samples taken, mean time of decompression was 13,14±16,68 months. The shortest decompression time applied was 1 month and the longest 60 months (Table 1).

**Table 1:**
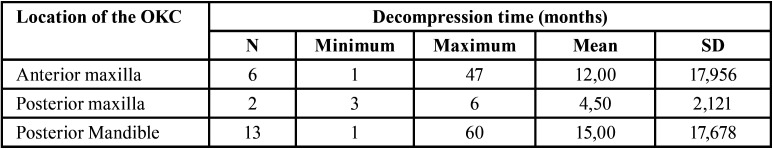
Decompression time depending on OKC location.

-Histopathological changes

Loss of parakeratinization in some area of the cystic epithelium was observed before decompression (4 cases). However, after surgical decompression, only 8 cases (38,1%) preserved a parakeratinized epithelium, while in 13 cases (61,9%) there was loss of parakeratinization in various areas or in the entire cystic epithelium. Loss of superficial parakeratinization was statistically significant after surgical decompression (*p*=0,007) (Table 2).

**Table 2:**
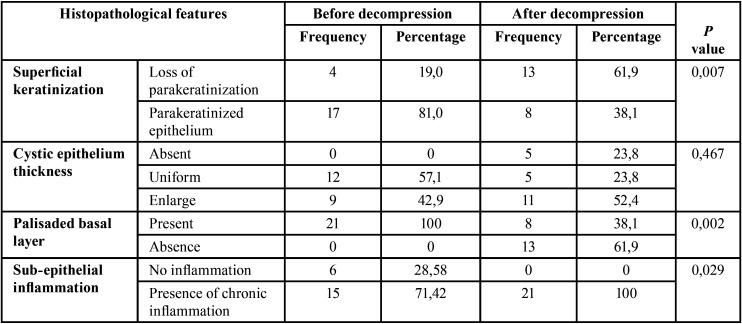
Summary of OKC histopathological changes before and after decompression.

Presence of atypical mitosis was only observed in one pre-decompression case. Epithelial mitosis was not observed in post-decompression cases.

The cystic epithelium thickness of pre-decompression samples was uniform in 12 cases (57,1%) and augmented in 9 (42,9%) cases. On the contrary, in the post-decompression samples we observed uniform thickness only in 5 samples (23,8%), and epithelium hyperplasia in 11 (52,4%). Absence of epithelium was observed in 5 cases (23,8%). When results were compared, differences were not statistically significant (*p*=0,467) (Table 2).

The palisaded basal layer was observed in all samples before decompression, but it was absent in the 61,9% of the cases after the surgical decompression. Statistically significant loss of the palisade was found after decompression (*p*=0,002) (Table 2).

Finally, chronic inflammation was observed in most of pre-decompression cases (6 cases were mild, 6 cases moderate and 3 cases showed intense inflammation). In contrast, all post-decompression cases showed inflammation (6 cases with mild, 8 moderated and 7 cases with intense sub-epithelial inflammation) (*p*=0,029) (Table 2).

-Cell proliferation 

**Figure 1:**
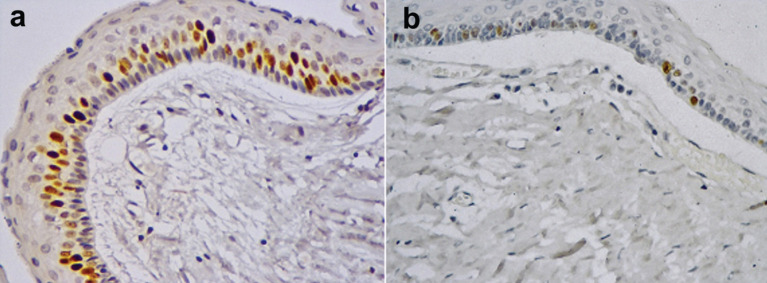
Immunoexpression of Ki-67 in the odontogenic keratocyst lining epithelium. A. Pre-compression cystic epithelium. Post-compression cystic epithelium. 400x.

Among pre-decompression samples, mean of Ki-67+ was 44,32±36, 72 cells. Interestingly, Ki-67 positive cells slightly decreased after decompression (31, 82±39, 73 cells) However, this reduction was not statistically significant (*p*=0,323), (Fig. 1).

**Figure 2:**
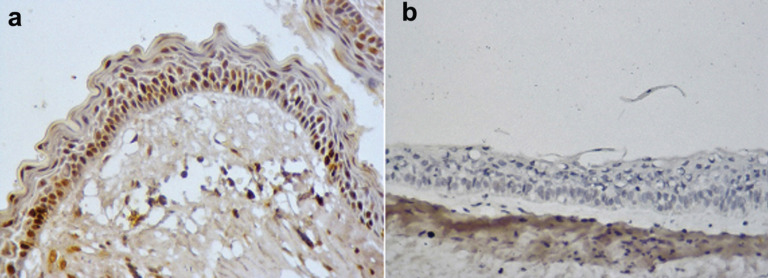
Immunoexpression of MCM4 in the odontogenic keratocyst lining epithelium. A. Pre-compression cystic epithelium. Post-compression cystic epithelium. 400x.

Average of immuno-positive stained for MCM4 was 32,12±38,33 cells in the pre-decompression group. Similar to Ki-67 results, the post-decompression group showed a decreased mean of 15,60±27,56 MCM4 positive cells, however, not statistically significant (*p*=0,079), (Fig. 2).

**Figure 3:**
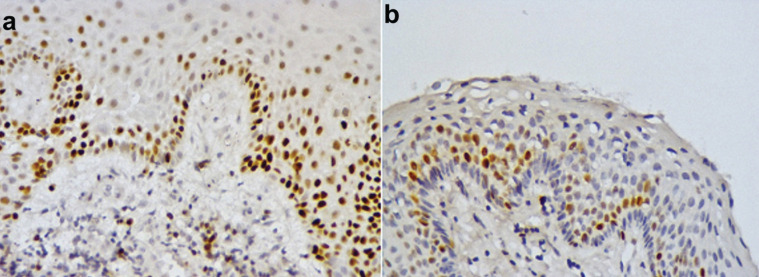
Immunoexpression of MCM7 in the odontogenic keratocyst lining epithelium. A. Pre-compression cystic epithelium. Post-compression cystic epithelium. 400x.

MCM7 expression was evaluated only in 17 samples. Among the pre-decompression samples there was a mean of 26,96±35, 74 positive cells. Diminution of MCM7+ cells were also observed in post-decompression cases (mean 19,27±29,57 cells). Again, decreased expression of MCM7 was not statistically significant (*p*=0,383), (Fig. 3).

-Apoptosis

**Figure 4:**
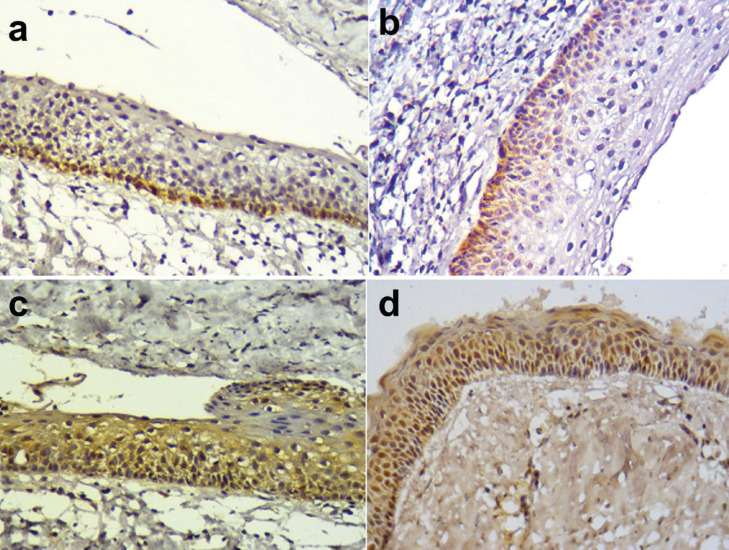
Immunoexpression of Bcl-2 and Bax in the odontogenic keratocyst. A; Bcl-2 positivity the pre-compression cystic epithelium. B; Bcl-2 positivity in the Post-compression cystic epithelium. C; Bax positivity the pre-compression cystic epithelium. D; Bax positivity in the Post-compression cystic epithelium. 400x.

Before surgical decompression, 16 cases showed Bax expression (5 showed a mild expression, 9 samples moderate and 2 samples an intense). 5 cases were negative. Similarly, after decompression treatment, 4 samples did not show any Bax staining and 17 cases were positive (5 samples showed a mild expression, 9 moderate and 3 samples intense) (Table 3). Bax results before and after decompression were similar (*p*=0,67), (Fig. 4).

**Table 3:**
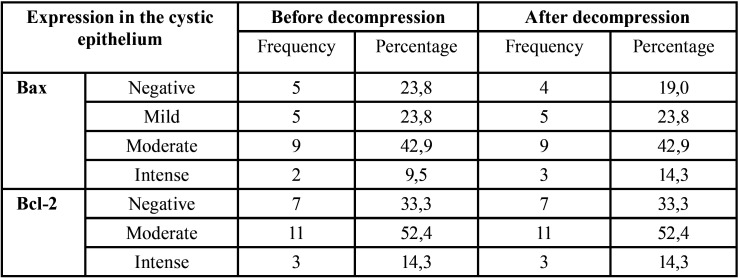
Epithelial Bax and Bcl-2 expression before and after decompression of the OKC.

The expression of Bcl-2 in the cystic epithelium was observed in 14 pre-decompression and 14 post-decompression samples. No changes were observed in Bcl-2 expression before and after decompression (Table 3).

## Discussion

OKC responds to conservative treatments such as surgical decompression, inducing modification of lining cystic wall until resemble normal oral mucosa (8,9). The role of surgical decompression, as a treatment for large pathologies, is widely known and accepted. It seeks to reduce the internal pressure of the cystic cavity, allowing the apposition of bone tissue and the reduction of the cyst dimensions that facilitates final surgical removal, minimizing the damage to adjacent anatomical structures and preserving patient’s function and esthetic (2-4).

Decompression time was arbitrarily determined by each clinician and varied widely in relation to the anatomical location of the cases. Interestingly it was observed that the longest time ranges were the cases located in the posterior mandibular area, this may be due to the fact that in this space, OKCs tend to reach larger dimensions without being noticeable or because the mandibular bone is denser, taking longer to regenerate, unlike the maxilla.

OKC presents a morphology that confers histopathological characteristics that differentiate it from other neoplasms or developmental cysts, features associated with its recurrence and aggressiveness potential. Epithelial features of the OKCs can be altered by decompression due to intense inflammation, to the point that the histopathological identification of the lesion may become more complex or even impossible (10).

Marker *et al*. (11) described after OKC decompression a modulation of the epithelium to a non-keratinized surface, associated with a decrease of aggressive behavior and increase in subepithelial inflammation. August *et al*. (8) established that the inflammatory process is precisely the one that leads to epithelial metaplastic changes, as well as the transformation of the OKCs epithelium to a non-keratinized one with an appearance similar to normal oral mucosa.

Partial or total loss of parakeratinization, decrease in the basal distribution of palisade cells, epithelial hyperplasia and an increase in capsular fibrosis induced by inflammatory changes are histopathological changes that allow, in decompressed OKC, an easier separation of the cystic wall from the bone tissue in a second phase, reducing the possibility of recurrence associated with the permanence of the remaining cystic tissue within the surgical area and facilitating the final treatment (8-12).

Cottom *et al*. (13) described a subepithelial separation and the presence of hyalinization in recurrent OKC cases, suggesting a substantial role for the basement membrane in the cellular cyst behavior. Changes in the basement membrane were not observed in the present study.

Regarding cell proliferation, a discrete decrease in the expression of Ki-67 and MCM4/7 was observed in OKC after surgical decompression in our study, suggesting a reduction of proliferative activity and therefore an apparently less aggressive behavior, probably by detection of aberrant cell growth and/or multiplication.

Nakamura *et al*. (9) coincided with the results described, after comparing the proliferative activity of epithelial cells through the expression of Ki-67 in 28 patients diagnosed with OKCs before and after marsupialization, observing decrease Ki-67+ cells after treatment, although not statistically significant. Likewise, Awni *et al*. (14) and Clark *et al*. (15) found no cell proliferation difference before and after surgical decompression.

To our knowledge there are no publications to date using of MCM4 or MCM7 as a cell proliferation marker in OCK before and after surgical decompression. Acharya *et al*. (16) and Jaafari-Ashkavandi *et al*. (17) both report MCM2-7 complex as cell proliferation marker, even more sensitive than Ki-67 according to the results of their investigations after comparing the expression of both markers in OKC, ameloblastoma and dentigerous cyst. MCM2-7 proteins have an essential role in the initiation and regulation of the DNA replication and may be used to distinguish cells that are in aberrant cell proliferative activity. MCM proteins can be detected, unlike Ki-67, from the early G1 phase of the cell cycle, being able to even express itself in those non-proliferative cells that are waiting to enter in the cycle, which for Ki-67 might pass undetected (18).

On the other hand, cell death can be assessed through the expression of the Bcl-2 family proteins, they have the ability to regulate the apoptosis process through pro and anti-apoptotic proteins such as Bax and Bcl-2 respectively. An increase in the detection of Bax favors cell death, while an excess in the expression of Bcl-2 prolongs cell survival by inhibiting apoptosis, promoting the development of a pathological lesion. The positivity of both markers in OKC has been previously reported independently, coinciding with a high expression of Bcl-2 that the authors correlate with the aggressive clinical behavior of the cyst (19-26).

Soluk Tekkeşın *et al*. (24) evaluated the expression of Ki-67, Bax and Bcl-2 in 60 cases of radicular cysts, OKCs and ameloblastomas, finding in the last two lesions a high expression of Ki-67 and Bcl-2, counteracted to a low expression of Bax. Their results coincide with those reported by Naz *et al*. (21) who also evidenced Bcl-2 was expressed even more in recurrent lesions.

In contrast, Tenorio *et al*. (23) and Rangiani *et al*. (25) reported in OKCs a low expression of Bcl-2 extended to the basal cell layer of the epithelial lining, while Bax showed a diffuse weak labeling. Lack of expression in the epithelial upper layers may be due to the decrease in the cell ability to divide.

Study of Diniz *et al*. (26) is the only work found that evaluates markers related to cell death before and after surgical marsupialization, reporting in 18 OKCs samples a decrease in the expression of Bcl-2, followed by an increase in miR-15a/16-1 (Bcl-2 genetic modulator). Marsupialization was capable of modify the anti-apoptotic rate, reducing its capacity for cell survival, and possibly its aggressive behavior.

No changes of Bax and Bcl-2 were observed in our study. The recovery or maintenance of the balance between pro and anti-apoptotic cells during or after surgical decompression, may suggest slowing of cyst growth, which with other factors contributes to its involution and therefore decompression success. To our knowledge, there are not previous studies that compare the expression of Bax in OKC before and after surgical decompression.

OKC treatment should be aimed to those modalities that eradicate the lesion with a low risk of recurrence at a low morbidity rate (2-4), evaluating not only the behavior of the cyst by itself, but also its response to the different techniques and their stability over time. The selection of surgical treatment should considerate clinical factors like patient’s age, anatomic location and dimensions of the lesion and, if it is primary or recurrent, allowing the surgeon to individualize each case as required. As the present study shows, surgical decompression allows a conservative management of OKC in a primary presentation, generating histological structural changes that can apparently reduce or stop its aggressive behavior and/or cell growth, in addition to the clinical thickening of the cystic capsule, which will facilitate its total excision with favorable results.

## Conclusions

Surgical decompression generates histological structural changes in OKC both in the epithelium and in the conjunctive wall generated by inflammation; however, these findings do not seem to alter the induction of the cell cycle or the rate of epithelial apoptosis.
